# Extremity injuries and dementia disproportionately increase the risk for long-term care at older age in an analysis of German Health Insurance routine data for the years 2006 to 2010

**DOI:** 10.1186/s11556-016-0169-8

**Published:** 2016-12-06

**Authors:** Alexander Barth, Anja Vatterrott, Ying Zhou, Anne Fink, Gabriele Doblhammer

**Affiliations:** 1 Empirical Social Research and Demography, Institute of Sociology and Demography, University of Rostock, Ulmenstr. 69, 18057 Rostock, Germany; 2 German Center for Neurodegenerative Diseases, Rostock, Germany; 3 Rostock Center for the Study of Demographic Change, Rostock, Germany; 4 German Center for Neurodegenerative Diseases, Bonn/Rostock, Germany

**Keywords:** Extremities, Mobility limitation, Dementia, Long-term care, Aged

## Abstract

**Background:**

Extremity injuries (EI) and dementia are important causes of long-term care (LTC), but they can also cause each other and are often present concurrently. Mobility-limiting EI can increase the risk of dementia, and dementia increases the risk for falls, which are often the cause of EI. When EI and dementia are present together, they can increase their negative effect on long-term care risk. This study aims to assess the strength of this interaction and the role of different body regions and severities of EI regarding LTC risk.

**Methods:**

We use Cox proportional-hazard models on LTC as dependent variable. EI (primarily fractures) and dementia (all types) are the central independent variables. We control for age, sex, rehabilitation and 18 relevant comorbidities. Analyses are based on health claims records for 2004–2010 for a random sample of about 122.000 insurants of Germany's largest public health insurance "AOK" aged 65+, about 25.000 of whom entered LTC.

**Results:**

Without concurrent dementia, non-severe EI (NSEI) of the lower and both extremities and all kinds of severe EI (SEI) increase LTC risk (HR: hazard ratio with 95% confidence interval. Lower NSEI: HR = 1.09 [1.05–1.14]; both NSEI: HR = 1.36 [1.29–1.44]. Lower SEI: HR = 1.67 [1.57–1.79]; upper SEI: HR = 1.27 [1.19–1.37]; both SEI: HR = 1.94 [1.81–2.07]). Dementia alone increases LTC risk more than fourfold (HR = 4.23 [4.11–4.35]).

Taking the interaction of EI and dementia into account, the concurrent presence of EI and dementia tends to increase the LTC risk more than expected for lower as well as upper NSEI and SEI. Summarily, when lower or upper EI and dementia are both present, the LTC risk tends to be higher than expected, suggesting synergistic effects.

**Conclusions:**

EI and dementia are important independent risk factors for long-term care. When lower or upper EI and dementia are present together, the resulting long-term care risk is increased disproportionately. Since the concurrent presence of both conditions increases the risk for care need, and a working treatment for dementia is not in sight, preventing EI, lessening the impact of EI and improving the outlook after an EI could help to reduce LTC need in the coming decades.

## Key points

Dementia and extremity injuries can increase each other’s impact on long-term care risk. The effect of dementia and simultaneous extremity injury increases the long-term care risk beyond that caused by dementia alone and more than expected, suggesting synergistic effects. The size of the combined risk depends on the body region and severity of the extremity injury. Severe extremity injury increases risk more than upper extremity injury, and lower extremity injury more than upper extremity injury. Targeted prevention and treatment of extremity injuries might help to reduce long-term care need in the future, even if a treatment for dementia is not forthcoming.

## Background

Extremity injuries (EI) due to falls [1, 2] are common among older persons [3]. Together with dementia [4], they are important causes of long term care (LTC) [5–8]. In western societies older age groups are growing due to rising life expectancy and large cohorts entering old age. Older people are more likely to experience falls and subsequently incur fractures than younger people [9] and to experience dementia. Increasing EI incidence has also been noted due to increasing fracture incidence independent of age structure, most likely because the number and severity of falls in older populations has increased [10]. In the future, even more people may be expected to experience and live longer with dementia, thus both risk factors might become even more important [11]. EI and dementia will continue to affect LTC need – a field already under considerable strain due to financial and personnel shortages. Not much is known about the combined effects of EI and dementia on LTC, especially in terms of a finer distinction of severe and non-severe injuries of the lower, upper or both extremities. EI and dementia are discrete LTC risks, but can also be causally related and are often present together [12–14].

Maintaining an active and regular social life as well as regularly performing productive tasks decreases the risk of dementia [15], because the associated cognitive stimulation helps preserve cognitive functioning. Lower and upper EI can drastically reduce the ability to uphold social interaction or perform productive tasks at the usual levels and act as a risk factor for dementia in that they reduce cognitive stimulation [16, 17]. Lower EI that limit mobility might be even more influential. Thus, the onset of mobility problems, such as unsteady gait, is recognized as a valid predictor of later cognitive decline [14, 18, 19]. An EI can also indirectly increase the risk for dementia due to a subsequent delirium, which is a risk factor for dementia as well [20].

Conversely, dementia is a pre-existing condition for many fractures, because cognitive deterioration also affects gait and balance, increasing the risk of falling [9, 14, 21, 22]. Dementia thus leads to earlier or more frequent falls and may also increase the severity of fall-related injuries. If present concurrently, the complementary effect of the two central LTC risk factors EI and dementia might further compound the LTC risk. However, the strength of their synergistic effect and to what degree the body region and severity of an EI influence the resulting LTC risk remains unclear.

Our study addresses four topics. First we differentiate between severe and non-severe injuries and hypothesize that Severe EI (SEI) leads to higher LTC risk than Non-Severe EI (NSEI). Second, we distinguish between lower, upper, and both extremities. Lower EI affect movement, upper EI affect manipulation of the environment, and a combination of both might differently affect LTC risk on their own or in conjunction with dementia. Moreover, both kinds of EI may indicate either one drastic injury that affects both extremities or multiple incidents. Third, as limited mobility may reduce cognitive activity and mobility, mobility-limiting lower EI and dementia should increase LTC risk more than upper EI and dementia, with SEI generally causing a higher risk than NSEI. Fourth, we explore the combined effects of dementia and EI on LTC, expecting them to be larger than the individual effect sizes simply added up [23].

## Methods

### Data

We use health claims data covering a random sample (*N* = 250.000) of insurants from ages 50 and higher of Germany's largest public health insurance, AOK ("Allgemeine Ortskrankenkasse"). AOK covers one in three citizens aged 50+, with an even higher share at older ages where it reaches 50% among the oldest old. As health insurance is mandatory, only few citizens (less than 0.3%) are not covered by any health insurance, 11% of the population is covered by private health insurance. Over all ages combined, the AOK sample differs from the German population in terms of age structure and socio-economic status. However, the differences become small at the highest ages where dementia is most prominent. Additionally, the age-specific death rates from our data indicate that mortality in AOK data fits well with the mortality of the whole German population, as another study using the same data shows [24]. An age-stratified sample was drawn based on all insurants in the first quarter of 2004 who were born in or before 1954, regardless of seeking treatment. Longitudinal observation takes place from 2004 to 2010 and contains one spell per quarter. The study period covers 2006–2010. The two preceding years are used to verify dementia diagnoses by means of multiple diagnoses by different specialists or over time. Thus all prevalent dementia diagnoses from 2006 onwards are validated.

Claims data are process-generated, detailed, and comprehensive. They contain all inpatient and outpatient diagnoses as ICD-10-codes, diagnosing specialist, prescriptions, rehabilitations with prognosis, costs, dates of birth and death, and sex. Only those diagnoses relevant for treatment and refundable by the insurer are recorded. All population groups are covered, including groups such as institutionalized persons in LTC, who are often subject to EI or dementia and missing in other studies. Although common survey problems such as selectivity or recall uncertainty are avoided, variables such as socioeconomic status or the severity of dementia are not available.

### Case count and variables

Cleaning, consistency checking, and especially the reduction to those at least 65 years old left 156.527 persons/770.000 person-years. As our outcome variable is the incidence of LTC, we removed all persons who were already in LTC in 2004–2005.

### Dependent variable: long term care entry

We define individuals who received benefits from Germany's statutory long-term care insurance as being in need of LTC. The LTC insurance ("Pflegeversicherung") was established in 1995 as a pay-as-you-go scheme, just like other branches of Germany's social security system (e.g. health care, unemployment). LTC benefits are granted when an applicant passes an objective assessment primarily focused on ADL impairments and is assigned to one of three LTC levels that denote the amount of care required. The lowest level is granted when at least one and a half hours of care are required per day, at least half of which concern basic tasks. The highest level is granted when at least five hours of care are required (four of which concerning basic tasks). In our study, we do not distinguish the different LTC levels. The LTC variable is binary; it has and retains the value one from the time any level of LTC is first acquired.

### Independent variables: extremity injuries, dementia and comorbidities

EI are differentiated by body region and severity using diagnostic data of fractures and injuries of the upper and lower extremities, including the pelvis (ICD-10 codes S40–S99, relevant parts of T for wounds, luxations, contusions, burns, frostbites and amputations). Rehabilitation data allow us to identify severe EI cases, which are cases of EI who concurrently or later received a medium or long term rehabilitative measure prescribed specifically due to constraints in relevant activities of daily living or multistructural functional damages related to EI. We do not distinguish between different methods of rehabilitation and rehabilitative measures prescribed due to other conditions than EI are not considered. This allows for the differentiation of six EI types: Non-Severe Extremity Injuries (NSEI), which are classified as (1) lower NSEI for fractures and injuries from the foot up to the hip and also the pelvis, (2) upper NSEI for fractures in the hands, arms and shoulders, and (3) lower and upper NSEI when both lower and upper extremities are affected); and Severe Extremity Injuries (SEI) of the (4) lower, (5) upper, or (6) both extremities. Multiple injuries of the same extremity over time are not considered separately. Pelvic injuries are included as lower extremity injuries because they are common fall-related injuries and are comparable to other lower limb injuries in terms of their consequences [25].

Dementia is defined as one or more of the following diagnoses: Alzheimer's disease (ICD-10 codes F00/G30), vascular dementia (F01), Lewy body dementia (G31.82), circumscribed brain atrophy (G31.0), dementia as a side-effect of another disease, e.g. Parkinson's disease (F02, F05.1, G23.1), as well as other/not specified dementia (F03). To ensure validity, we considered only diagnoses flagged as "secure diagnosis" for outpatients or "discharge diagnosis" for inpatients. Second, at least two concurrent or later diagnoses either by an inpatient and an outpatient physician or from different outpatient specialists were required. If the first dementia diagnosis was given in the last quarter of the observation period, it was deemed valid.

Age is a categorical variable in 5 year-groups starting at ages 65–69, with the last age category 95 years and older. Chronic diseases and other ailments are included as binary variables taking the value one from the first occurrence from 2004 onwards and zero otherwise. They are: hypertension (relevant parts of I10–I15), diabetes (E10–E14), ischemic diseases (I20–I25), cerebral diseases (I60–69), hypercholesterolemia (E780), atrial fibrillation (I48), heart insufficiency (I50), lung insufficiency (J44), nervous diseases except Parkinson's disease and dementia (G20–G22), Parkinson's disease (G20–G22), gastric diseases (K0–K9), alcoholic liver disease (K70), atherosclerosis (I70), pneumonia (J12–J18), cases of infections or parasites (A-B), other external injuries (S-T, V-Y except related to EI), and smoking as well as non-smoking related cancers. All variables and corresponding occurrences, person-year exposures, and transition rates into LTC are shown in Table 1.

**Table 1:** Sample overview of long term care entry cases by age, sex, extremity injuries, rehabilitation, dementia and comorbidities

	Exposure (person years)	Cases	Incidence rate p. 100 PY	95% CI	
Total	510,957	25,150	4.9	4.9	5.0
Age groups					
65–69	93,189	1213	1.3	1.2	1.4
70–74	167,840	3641	2.2	2.1	2.2
75–79	125,332	5242	4.2	4.1	4.3
80–84	80,730	6781	8.4	8.2	8.6
85–89	34,889	5584	16.0	15.6	16.4
90–94	7485	2090	27.9	26.8	29.1
95 +	1491	599	40.2	37.1	43.5
Sex					
Male	200,175	8592	4.3	4.2	4.4
Female	310,782	16,558	5.3	5.2	5.4
No EI	368,033	16,058	4.4	4.3	4.4
Lower NSEI	48,770	2781	5.7	5.5	5.9
Upper NSEI	41,947	2130	5.1	4.9	5.3
Lower & upper NSEI	17,286	1455	8.4	8.0	8.9
Lower SEI	12,324	972	7.9	7.4	8.4
Upper SEI	13,677	816	6.0	5.6	6.4
Lower & upper SEI	8920	938	10.5	9.9	11.2
Rehabilitation	451,824	20,268	4.5	4.4	4.5
Yes	59,133	4882	8.3	8.0	8.5
Dementia	484,204	17,939	3.7	3.7	3.8
Yes	26,753	7211	27.0	26.3	27.6
Hypertension	102,676	6103	5.9	5.8	6.1
Yes	408,281	19,047	4.7	4.6	4.7
Diabetes	336,051	15,158	4.4	4.4	4.6
Yes	174,905	9992	5.7	5.6	5.8
Ischemic stroke	308,858	13,257	4.3	4.2	4.4
Yes	202,098	11,893	5.9	5.8	6.0
Cerebral diseases	398,693	15,270	3.8	3.8	3.9
Yes	112,264	9880	8.8	8.6	9.0
Hypercholesterolemia	347,585	18,762	5.4	5.3	5.5
Yes	163,372	6388	3.9	3.8	4.0
Atrial fibrillation	442,155	18,481	4.2	4.1	4.2
Yes	68,801	6669	9.7	9.5	9.9
Heart insufficiency	391,477	13,800	3.5	3.5	3.6
Yes	119,480	11,350	9.5	9.3	9.7
Lung insufficiency	429,475	20,109	4.7	4.6	4.7
Yes	81,482	5041	6.2	6.0	6.4
Nervous diseases	288,980	11,675	4.0	4.0	4.1
Yes	221,976	13,475	6.1	6.0	6.2
Parkinson's disease	500,037	23,519	4.7	4.6	4.8
Yes	10,919	1631	15.0	14.2	15.7
Gastric diseases	181,926	8846	4.9	4.8	5.0
Yes	329,031	16,304	5.0	4.9	5.0
Alcoholic liver disease	507,071	24,855	4.9	4.8	5.0
Yes	3886	295	7.6	6.8	8.5
Atherosclerosis	434,115	19,991	4.6	4.5	4.7
Yes	76,841	5159	6.7	6.5	6.9
Pneumonia	477,083	20,980	4.4	4.3	4.5
Yes	33,874	4170	12.3	11.9	12.7
Infections/Parasites	282,266	11,997	4.3	4.2	4.3
Yes	228,690	13,153	5.8	5.7	5.9
External injuries	254,719	10,546	4.1	4.1	4.2
Yes	256,238	14,604	5.7	5.6	5.8
Smoking-related cancer	472,757	21,559	4.6	4.5	4.6
Yes	38,199	3591	9.4	9.1	9.7
Non-smoking r. cancer	462,228	21,415	4.6	4.6	4.7
Yes	48,729	3735	7.7	7.4	7.9

*PY* person years, *CI* confidence interval

Source: AOK claims data, own calculations

### Statistical analysis

We use Kaplan-Meier survival analysis and Cox proportional hazard models to study LTC risk depending on EI and dementia, adjusted for comorbidities and other controls. Calendar time of the study period (2006–2010) is used as underlying process time. Diagnoses and other status changes (except death, which is given by the month of death) are placed in the middle of the quarter in which they occur. If death and care entry occur in the same quarter, care entry was placed in the month before death. LTC entry is available quarterly, with the exception of 2006, where it is available only at the end of the year and was placed mid-year. All diagnoses, age group and care-level entry are treated as time dependent, sex is time constant. All medical diagnoses, including EI and dementia, indicate whether a certain disease or status was ever observed starting from 2004 onwards. They were coded as one and remained like that from that point onwards until the end of the observation period. Thus, they can be interpreted as the effect of ever having experienced the respective condition since the beginning of 2004. Individuals are followed until censoring or death, depending on which occurred first.

## Results

Within the period of study 25,150 individuals received a care level, which is an incidence rate of 4.9 (95% CI = 4.9–5.0) individuals per 100 person years (Table 1). Dementia patients have an incidence rate seven times higher than non-demented individuals, which makes it the strongest cause of LTC after age higher than 90 years. Individuals without EI have a lower incidence rate than patients with any kind of EI. Generally, NSEI patients have lower incidence rates than SEI patients, and patients with upper EI have lower incidence rates than patients with lower or both types of EI. Patients with severe upper and lower EI show a relative risk nearly 2.5 times higher than patients without EI. The transition into LTC increases with age; the incidence rate roughly doubles for each 5 year interval. Females have a slightly higher incidence rate than males, and patients who received rehabilitative treatment show nearly double the incidence rate of those who did not, indicating that these treatments are typically reserved for more serious cases.

**Fig. 1:**
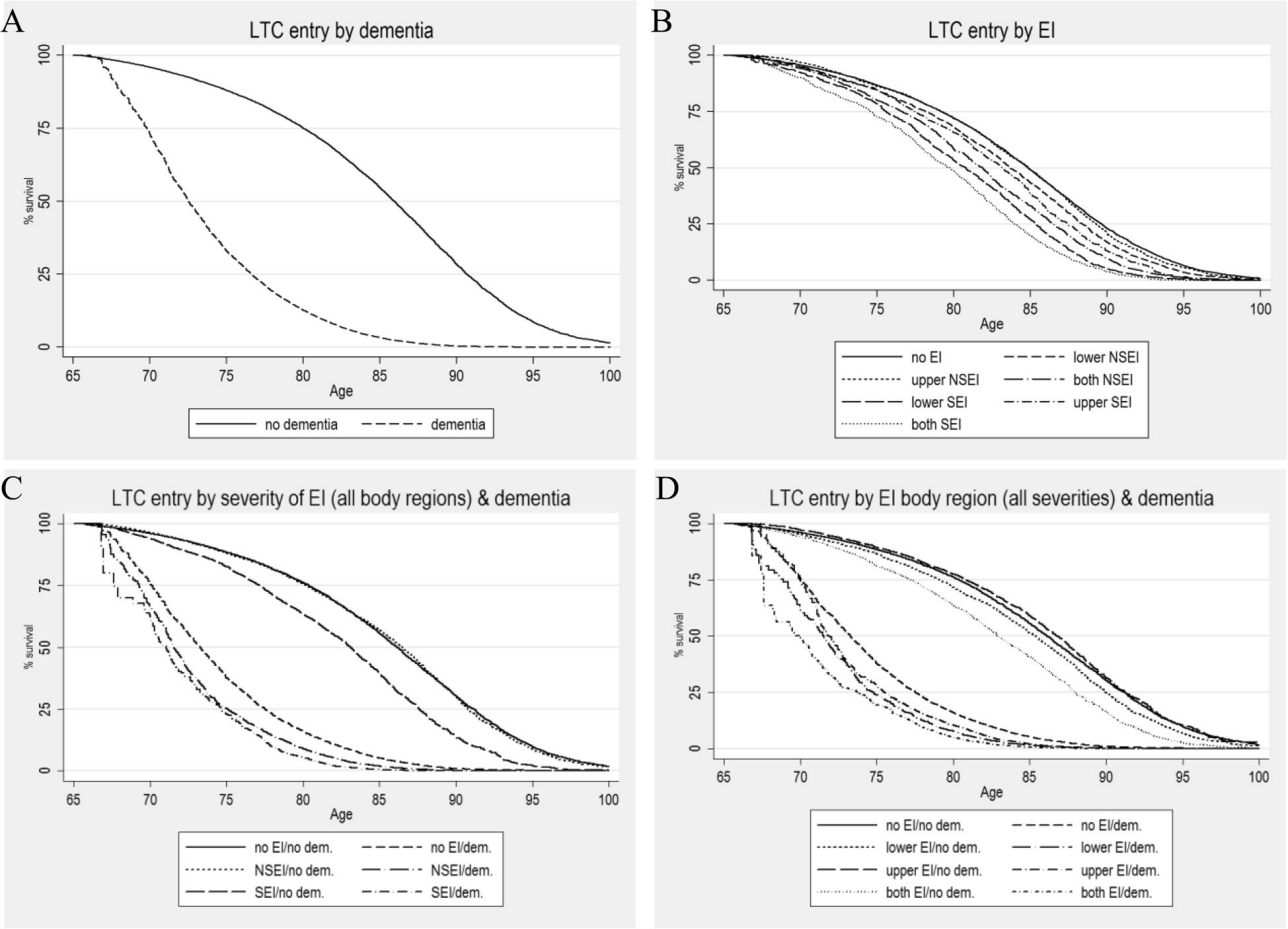
**a–d** Long term care entry over age by extremity injuries and dementia (Kaplan-Meier Survival Curves). Source: AOK claims data, own calculations

Dementia leads to faster LTC entry (Fig. 1a), as does EI. In particular, LTC risk increases from no to non-severe to severe EI, whereby upper EI generally show lower risk than lower EI, and both EI show higher risk than upper or lower EI. There is one exception, namely upper SEI, which increase the LTC risk less than both NSEI (Fig. 1b). Without dementia, SEI is an important risk factor for LTC, whereas NSEI does not differ from no EI (Fig. 1c). Compared to dementia alone, NSEI and SEI with dementia are associated with faster LTC transition. The body region affected by an EI plays an important role for LTC transition. Without dementia, especially both and lower EI increase LTC transition compared to upper and no EI. Compared to no EI with dementia, both EI and, to a smaller extent, lower and upper EI, increase the LTC transition (Fig. 1d). Log-rank and Wilcoxon tests generally indicate highly significant differences between the survivor functions.

Hazard ratios estimated by multivariate Cox proportional hazard models are presented in Table 2. The first model shows the main effects of EI, dementia, age and sex. The second model presents the EI-dementia interaction. Comorbidities and rehabilitation are added in model 3.

**Table 2:** Hazard ratios of long term care entry by age, sex, extremity injuries, rehabilitation and dementia

	Model 1^a^				Model 2^b^			Model 3^c^		
	HR	95% CI			HR	95% CI		HR	95% CI	
Age										
65–69 (ref.)	1.00				1.00			1.00		
70–74	1.62	1.51	1.73		1.62	1.52	1.73	1.56	1.47	1.67
75–79	2.89	2.72	3.08		2.90	2.72	3.09	2.65	2.49	2.83
80–84	5.25	4.93	5.58		5.26	4.95	5.60	4.53	4.26	4.83
85–89	8.96	8.41	9.55		8.99	8.43	9.58	7.51	7.04	8.01
90–94	14.29	13.30	15.36		14.32	13.32	15.39	11.51	10.69	12.39
95 plus	18.91	17.12	20.89		18.87	17.08	20.84	15.46	13.98	17.10
Male (ref.)	1.00				1.00			1.00		
Female	0.90	0.88	0.92		0.90	0.88	0.92	1.05	1.02	1.08
No rehabilitation (ref.)								1.00		
Rehabilitation								1.50	1.45	1.55
No dementia	1.00									
Dementia	4.23	4.11	4.35							
No EI										
	1.00			No dementia	1.00			1.00		
				Dementia	3.92	3.77	4.07	3.30	3.17	3.43
NSEI										
Lower	1.09	1.05	1.14	No dementia	1.03	0.98	1.09	0.98	0.93	1.03
				Dementia	4.87	4.57	5.19	3.91	3.66	4.17
Upper	0.97	0.92	1.01	No dementia	0.86	0.81	0.91	0.83	0.78	0.88
				Dementia	4.74	4.42	5.09	3.71	3.46	3.99
Both	1.36	1.29	1.44	No dementia	1.34	1.25	1.43	1.22	1.14	1.31
				Dementia	5.67	5.22	6.16	4.23	3.89	4.60
SEI										
Lower	1.67	1.57	1.79	No dementia	1.63	1.50	1.76	1.35	1.25	1.46
				Dementia	7.08	6.33	7.92	5.00	4.47	5.60
Upper	1.27	1.19	1.37	No dementia	1.19	1.09	1.29	1.11	1.02	1.21
				Dementia	5.97	5.26	6.77	4.85	4.27	5.51
Both	1.94	1.81	2.07	No dementia	1.95	1.79	2.11	1.63	1.50	1.77
				Dementia	7.73	6.92	8.63	5.06	4.52	5.66;

^a^Model 1 includes extremity injuries and dementia as individual variables and no interaction effect

^b^Models 2 and 3 include extremity injuries and dementia as interaction effect

^c^Model 3 controls for comorbidities (see Appendix for multivariate results)

Source: AOK claims data, own calculations

### Extremity injuries

For all body regions, SEI increase LTC risk more than NSEI (Table 2, Model 1), e.g. lower SEI are associated with a hazard ratio (HR) of 1.67 (95% CI: 1.57–1.79) and lower NSEI with a HR of 1.09 (95% CI: 1.05–1.14). Both EI together cause a higher LTC risk than EI of only one body region for NSEI (HR = 1.36, 95% CI: 1.29–1.44) and SEI (HR = 1.94, 95% CI: 1.81–2.07). Upper NSEI show no significant effect on LTC risk. Dementia is a distinct and strong LTC risk factor (HR = 4.23, 95% CI: 4.11–4.35).

### Extremity injuries with concurrent dementia

To assess the interaction between EI and dementia, the observed joint effects (Model 2) are compared with the expected joint effects (based on Model 1). The LTC risk resulting from the concurrent presence of EI and dementia tends to be greater than the expected risk resulting from lower and upper NSEI as well as SEI, and slightly smaller than expected for NSEI and SEI of both extremities at once. For instance, the expected joint effect of upper NSEI with concurrent dementia is HR = 4.1 (HR dementia * HR upper NSEI: 4.23 * 0.97), while the observed joint effect is HR = 4.74. Thus, for NSEI as well as SEI, the concurrent presence of dementia affects the LTC risk resulting from lower or upper EI, while it does not increase the LTC risk when both upper and lower extremities are injured.

Without concurrent dementia, the same pattern as in model 1 emerges regarding the role of EI severity. SEI of a given body region show higher LTC risk than NSEI in the same region, e.g. lower SEI with HR = 1.63 (95% CI: 1.50–1.76) and lower NSEI with HR = 1.03 (95% CI: 0.98–1.09, not significant). Interestingly, upper NSEI without dementia even show a protective effect (HR = 0.86, 95% CI: 0.81–0.91), but when dementia is present concurrently, LTC risk is increased, as the resulting joint effect (HR = 4.74, 95% CI: 4.42–5.09) is higher than that of dementia alone.

Dementia alone remains an important LTC risk factor, as it increases LTC risk nearly fourfold (HR = 3.92, 95% CI: 3.77–4.07). Summarily, LTC risk is higher for all kinds of EI if dementia is present as well, and also tends to be higher than expected in the cases of lower or upper NSEI and SEI.

### Full model including comorbidities

Finally, adding comorbidities (see Appendix) and rehabilitation reduces the effect sizes of EI and dementia, but the relation of different kinds and severities of EI and dementia to each other that emerged in the previous models remains unchanged (Model 3). For example, lower SEI with dementia (HR = 5.00, 95% CI: 4.47–5.60) show a higher relative risk than lower NSEI with dementia (HR = 3.91, 95% CI: 3.66–4.17). The comorbidities show mostly higher LTC risk, with the exceptions of gastric diseases, ischemic stroke, hypertension, and hypercholesterolemia. Rehabilitative treatment is associated with higher LTC risk (HR = 1.50, 95% CI: 1.45–1.55) and seems to be prescribed to people who are more likely to end up in LTC.

## Discussion

We show that concurrent with dementia, NSEI and SEI increase the LTC risk above the level associated with dementia alone. Moreover, when concurrent with dementia, upper or lower non-severe and severe EI tend to show LTC risks that are larger than expected. Because both afflictions are common comorbidities that can influence or strengthen each other, this fits in with previous work. Even without such interaction, EI and dementia are two central independent LTC risk factors. While confirming the importance of dementia on LTC, we also show that SEI increase LTC risk more than NSEI, and among both degrees of severity, upper EI contribute less risk than lower or both SEI. EI can severely limit basic self care capabilities, e.g. transfer within the home [26], hygiene, or food preparation, which are qualifiers for LTC benefits. Only lower SEI, which could affect transfer within the home, show increased LTC risk. Moving to where a specific task should take place is a necessary prerequisite of performing said task, thus EI limiting mobility can be seen as a cause for LTC on a more basic level than upper EI limiting interaction. LTC risk linked to upper EI is a significant influence only when the EI is severe, or when concurrent with dementia. Dementia can, depending on its progress, necessitate LTC because it constrains the ability to live independently on many levels [5, 7]. Lower EI are not only problematic in itself, as our results show, but are also a prominent cause for decline in physical activity, which has been linked to a higher occurrence of dementia [12, 13]. Lower EI can impede social interaction and, by extension, cognitive activity [18, 19]. Dementia is often a pre-existing condition for falls and fractures [9], thus dementia patients sustain EI more often than those free of dementia. Keeping a balanced gait relies on the same brain area affected by dementia, thus explaining why, at older ages, mobility-limiting EI can often be traced back to cognitive deficits [21, 22]. This mutual influence might explain why individuals with dementia and EI show the highest LTC risk.

### Strengths and weaknesses

This study's main weaknesses are rooted in the data, which were collected in conjunction with the refunding of medical practitioners. Thus, dementia might be underreported, because only diagnoses related to an actual treatment are recorded. Thus, early stage dementia or severe dementia might not be documented at all times, because a treatment is not yet deemed reasonable or futile [11]. If dementia was only diagnosed once during the study time, it would not register as such in our data due to our reliance on the validation procedure that requires at least two diagnoses. Second, we chose to define EI in a strict sense. Other injuries that might be comparable to EI in terms of effects on mobility limitation are thus not classified as EI. Furthermore, not every SEI can be classified as such, because we rely on rehabilitation data, and available funds for rehabilitative treatments are limited. Thus, not every SEI case might receive such treatment. Only diagnoses relevant for treatment and refundable by the insurer are recorded. The definition of LTC is based solely on whether a person received benefits from the statutory LTC insurance. Not everyone in need of care might be classified as such in the data, because someone might not know about the availability of those benefits, or because the application process was deemed too difficult. Last, there might be some selectivity in the choice of private vs. public (such as AOK) health insurance and within the public sector. Even though AOK is Germany's largest public health insurance, our sample is not completely representative of adults older than 49 years, however, with age, the differences become smaller and the mortality in AOK data fits well with the mortality pattern of the whole German population [24].

The main strengths of this study are the claims data used. They are process-generated, detailed, and comprehensive. The outcome variable LTC benefit eligibility is diagnosed externally and objectively. The data are not subject to health-related self-selection beyond the choice of health insurance, because all people insured with AOK older than 50 in the first quarter of 2004 were eligible to be drawn into our sample. The data includes groups such as institutionalized persons in LTC that are often subject to EI or dementia. Other common survey problems, e.g. recall uncertainty, are avoided as well.

## Conclusions

Despite its' importance as a risk factor for care need, the interaction of EI and dementia has not been the explicit subject of many studies. Preventing EI can reduce LTC in two ways: for persons affected only by EI, and for persons in whom EI and concurrent dementia increase LTC risk beyond that associated with dementia alone. As a viable treatment for dementia is not expected to be forthcoming in the foreseeable future and as EI and dementia often occur together, preventing EI or improving their treatment could be a promising strategy to counter LTC need in ageing societies. Because mobility limitations have also been shown to cause or accelerate the onset of dementia [14], better EI prevention and rehabilitation may not only reduce the direct LTC risk, but may also help to prevent dementia. Special attention can be paid to exercises that promote regular activity and which train gait and balance, because they can help prevent falls and subsequent injuries in the first place, but evidence on their effectiveness is mixed and suggests that especially for frail people, exercise alone does not protect from falls, so focusing on the safety of mobility is of high importance. Otherwise, frail people might have a higher risk of falling with higher levels of mobility [27–29]. Even for dementia patients, multifactorial fall prevention programs [30] as well as cognitive-motor dual task exercises for individuals without cognitive impairments have been shown to be effective [31]. Innovative mobility-related assistive devices used primarily by older individuals already affected by ML provide another possibility [32]. Need-driven usage of mobility-related assistive devices is the typical case [33], suggesting the main objective should be offering adequate, user-friendly and effective devices, and their adoption by users should follow naturally. The introduction of such devices could even by financed by private copayments because, to a certain extent, there is willingness to effect individual copayments to purchase assistive devices [34]. The rehabilitative process also leaves room for improvements especially tailored for patients with cognitive impairments and EI. For instance, special post-operative, outpatient treatments for dementia patients with hip fractures by specialized personnel effectively helped cognitively impaired fracture patients to regain their pre-injury functional status [35]. If such a support chain were standard, better recovery of previous mobility status could be achieved, thereby reducing or postponing care risk.

## Appendix

**Table 3:** Hazard ratios of care need by comorbidities

	Model 3*		
	HR	95% CI	
Diseases (ref: not afflicted)			
Hypertension	0.51	0.49	0.52
Diabetes	1.15	1.12	1.18
Ischemic stroke	0.83	0.80	0.85
Cerebral stroke	1.42	1.38	1.46
Hypercholesterolemia	0.75	0.73	0.78
Atrial fibrillation	1.41	1.37	1.45
Parkinson's disease	1.56	1.49	1.65
Heart insufficiency	1.50	1.46	1.55
Pneumonia	1.80	1.74	1.87
Lung insufficiency	1.04	1.00	1.07
Nervous disease	1.10	1.07	1.13
Gastric disease	0.77	0.75	0.79
Alcoholic liver disease	1.87	1.66	2.09
Atherosclerosis	1.05	1.02	1.09
Infections/parasites	1.14	1.11	1.17
External injuries	0.97	0.95	1.00
Smoking related cancers	1.72	1.66	1.79
Non smoking related cancer	1.32	1.27	1.37

HR: hazard ratio, CI: confidence interval

*Model 3 controls for extremity injuries, dementia, age, sex and rehabilitation (see Table 2)

Source: AOK claims data, own calculations

## Abbreviations

EI: Extremity injury
LTC: Long-term care
SEI: Severe extremity injuries
NSEI: Non-severe extremity injuries
